# Oncolytic Adenoviruses for Cancer Therapy

**DOI:** 10.3390/ijms22052517

**Published:** 2021-03-03

**Authors:** Lorella Tripodi, Maria Vitale, Vincenzo Cerullo, Lucio Pastore

**Affiliations:** 1 SEMM European School for Molecular Medicine, 20123 Milano, Italy; tripodi@ceinge.unina.it; 2 CEINGE Biotecnologie Avanzate, 80145 Naples, Italy; vitalema@ceinge.unina.it; 3 Dipartimento di Medicina Molecolare e Biotecnologie Mediche, Università di Napoli Federico II, 80131 Napoli, Italy; 4 Laboratory of Immunovirotherapy, Drug Research Program, Faculty of Pharmacy, University of Helsinki, 00014 Helsinki, Finland

**Keywords:** oncolytic adenovirus, cancer, immunovirotherapy

## Abstract

Many immuno-therapeutic strategies are currently being developed to fight cancer. In this scenario, oncolytic adenoviruses (Onc.Ads) have an interesting role for their peculiar tumor selectivity, safety, and transgene-delivery capability. The major strength of the Onc.Ads is the extraordinary immunogenicity that leads to a strong T-cell response, which, together with the possibility of the delivery of a therapeutic transgene, could be more effective than current strategies. In this review, we travel in the adenovirus (Ads) and Onc.Ads world, focusing on a variety of strategies that can enhance Onc.Ads antitumoral efficacy, passing through tumor microenvironment modulation. Onc.Ads-based therapeutic strategies constitute additional weapons in the fight against cancer and appear to potentiate conventional and immune checkpoint inhibitors (ICIs)-based therapies leading to a promising scenario.

## 1. Introduction: A Journey in the Adenovirus World

The history of adenoviruses (Ads) begins in the 20th century when the first Ad was discovered and characterized [1]. Over the years, the relevance of research in the Ad field has increased, not only for the possibility to elucidate its pathogenetic mechanisms but also for the development of Ad-derived vectors for gene and cancer therapy [2,3,4,5]. Ads are non-enveloped, episomal, lytic DNA viruses with a 90 nm capsid and a genome of approximately 38 kb [6]. For its infectious properties, i.e., the ability to infect a large number of cell types, Ads-derived vectors have been extensively studied for gene therapy [3,6,7,8] and as anticancer agents [2,4,9,10]. The main advantages of using Ad vectors include easy genetic modification, the possibility of high titer production, and their physiochemical stability [6]. In addition, helper-dependent adenoviral (HD-Ad) vectors have a 38 kb capacity allowing the introduction of large transgenes [3]. These properties make them suitable for use as oncolytic viruses (OVs) with a few simple genetic manipulations. OVs were originally developed to destroy cancer cells selectively, with reduced harm to normal cells [11]. Lately, evidence has accumulated regarding the ability of OVs to induce an immune reaction against tumor cells overcoming tumor mechanisms of immune evasion; at present, this is considered the main mechanism of the antitumoral effect of OVs [12].

## 2. Adenovirus (Ads) Vector Design

Human adenoviruses (hAds) are nonenveloped viruses with a diameter of 70 to 100 nm. The external protein shell of the virus is icosahedral, with 20 triangular faces, 30 edges, and 12 vertices, and this symmetry is made up in large parts by the major virus protein, hexon. hAds are members of the family Adenoviridae and are classified into the genus *Mastadenovirus*. There are 51 human Ad serotypes originally classified based on their ability to be neutralized by specific animal antisera. These can be further subdivided into seven species—or subgroups—(A to G), with a further subdivision of species B into subspecies B1 and B2 on the basis of their capacity to clump erythrocytes of humans, rats and monkeys as well as on the basis of their oncogenicity in rodents. More than 30 simian adenoviruses (sAds) display sequence identities to their human counterparts to such an extent that they have also been included in the taxonomy of human adenoviruses, within species B, C, E, and G [13]. hAds were initially isolated mainly from military forces with acute febrile respiratory disease and were subsequently associated with a number of clinical signs, including keratoconjunctivitis, gastroenteritis, hepatitis, meningoencephalitis, cystitis, upper and lower respiratory tract infections, and myocarditis, but also with noninflammatory conditions, such as obesity [14]. hAds infections are easily transmittable and, in some instances, highly contagious. Although the clinical courses are usually mild and self-limiting, infections may cause localized outbreaks with a critical course, occasionally leading to a lethal outcome even in the immunocompetent [15].

**Figure 1:**
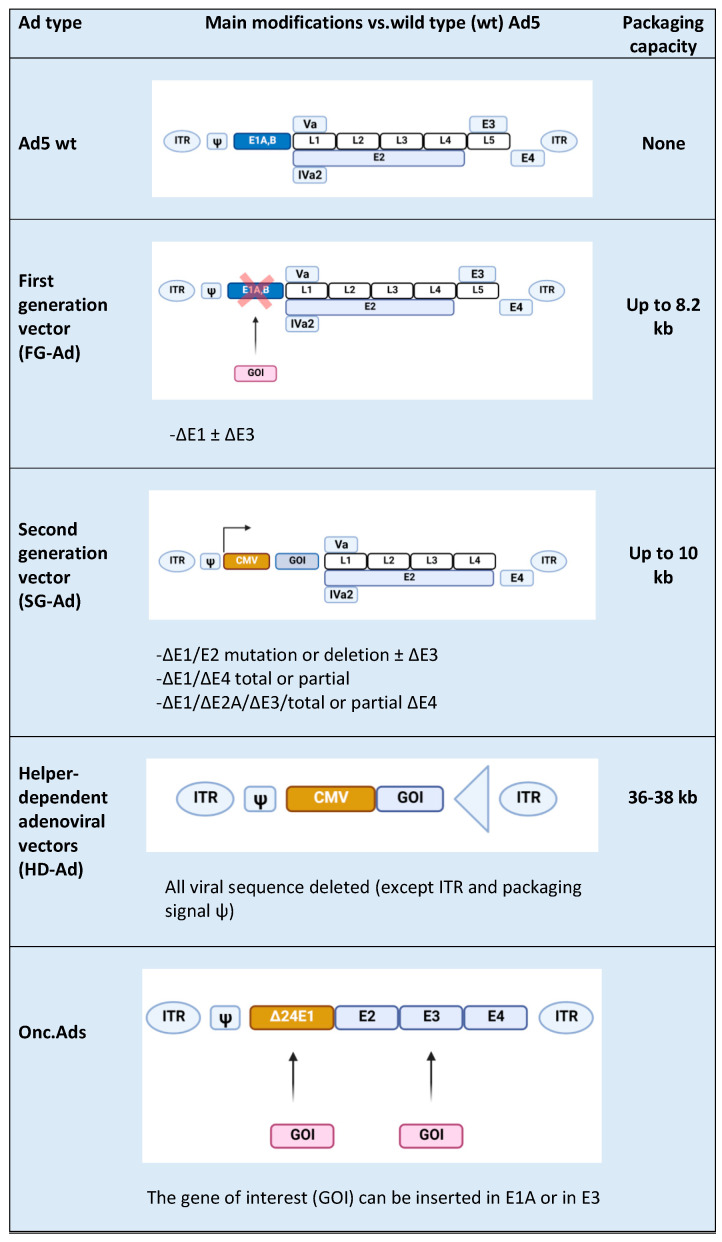
Types of Ad5-derived vectors and their characteristics: wild type (wt), first generation (FG-Ad), second generation (SG-Ad), and helper-dependent (HD-Ad) adenoviral vectors. At the end of the table is a schematic representation of an Onc.Ad. The figure was created with BioRender.com.

Ads are usually modified in specific regions [16], such as E1, E2A, E3, and E4 genes (Figure 1) [17]. E1, E2a, and E4 genes are essential for vector replications and are complemented in producer cell lines such as HEK-293 and their subsequently modified versions [17]; vectors with deletion of the above-mentioned genes are replication-defective but still maintain the ability to induce a strong host immune response towards both vectors and transgenes. The E3 gene is dispensable for vector replication and is deleted to increase vector capacity. Subsequent generations of Ad vectors have been developed leading to safer, less toxic, and more capable vectors. In first-generation adenoviral (FG-Ad) vectors, the E1 gene is deleted and replaced with the transgene; the packaging capacity of FG-Ad vectors is limited because only a limited amount of virus genome is deleted (8.2 kb) [18], and, consequently, the inserted transgene can be of limited size. Second-generation adenoviral (SG-Ad) vectors have deletions in E1, E3, and E2 or E4 genes, resulting in a reduced possibility of reversion to a replication-competent Ad and an increased room to accommodate larger transgenes (up to 10 kb). Finally, using helper-dependent adenoviral (HD-Ad) vectors, the whole genome can be substituted with DNA of interest. HD-Ad vectors contain only cis-acting Ads sequences necessary for viral DNA replication and a packaging and can accommodate up to 35 kb of foreign DNA [7,19]. Production of HD-Ad vectors requires a helper virus (HV) that provides all the protein products necessary for replication [20] expressing the Cre recombinase that eliminates the possibility of HV genome packaging. High accommodation capacity is one of the principal advantages of HD-Ads, together with the ability to efficiently transduce a wide variety of cell types, regardless of the cell cycle [21]. The complete absence of the viral genes attenuates cellular toxicity and host response, resulting in decreased virus clearance [22]; in addition, the minimal overlap with the adenoviral HV genome abrogates the possibility of generating wild-type (wt) Ad by recombination events.

Although Ads have been originally developed for transgene delivery in gene therapy applications, they have become interesting candidates as an anticancer agent. In fact, the development of replicative Onc.Ads to selectively replicate in cancer cells has been one of the early innovative strategies for cancer gene therapy [23]. Expression of E1A, a protein with a pivotal role in the early virus replicative cycle, under the control of a promoter typically overexpressed in specific cancer cells and usually not expressed in the normal tissues was the first strategy for cancer targeting [1]. A subsequent strategy to develop tumor-selective Onc.Ads consisted in the expression of a defective E1A unable to support replication in normal cells but able to lead to replication in the absence of the retinoblastoma protein (pRb) typically lacking in cancer cells. E1A-defective production is obtained with a deletion of 24 bp on this gene (E1A-d24) and the addition of an E2F promoter before the E1A-d24 gene [1].

The main feature of Onc.Ads that leads to their antitumoral effects consists in the ability to induce an immune response against cancer cells in different ways, both modulating the tumor microenvironment (TME) and through the release of tumor-associated antigens (TAAs) and neoantigens that are subsequently processed principally by antigen-presenting cells (APCs). In fact, Onc.Ads induce different types of immunogenic cell death (ICD), such as necrosis, necroptosis, pyroptosis, autophagic cell death, and immunogenic apoptosis. Although Onc.Ads have a significant effect on tumor cells’ growth and tumor size in vivo, they are usually unable to eradicate the whole tumor. In order to potentiate Onc.Ads effects, a local co-administration with HD-Ad vectors expressing a variety of antitumoral proteins revealed to be a promising strategy [20]. Indeed, the synergistic effect of Onc.Ads/HD-Ad vectors administration allows the amplification and packaging of HD-Ad vectors in cancer cells and overcomes the limitations of each virus: the reduced capacity of Onc.Ads and the inability of HD-Ad vectors to replicate in tumors [20].

## 3. Exploring the Tumor Microenvironment and Its Modulation by Onc.Ads

Since Rudolf Virchow discovered the presence of leukocytes in neoplastic tissues and proposed the link between chronic inflammation and tumorigenesis [24], a comprehensive understanding of the TME of solid tumors has attracted researchers’ attention [25]. The TME is a heterogeneous cellular environment in which the tumor propagates. Solid malignant tumors include not only tumor cells but also several non-transformed cells, including mesenchymal cells (cancer stem cells (CSCs), mesenchymal stem cells (MSCs), endothelial cells (ECS), fibroblasts, and myofibrobasts) that contribute to tumor cells’ growth. Often, the TME contains innate and adaptive immune cells including dendritic cells (DCs), mast cells (MCs), macrophages, neutrophils, T-cells, B-cells, natural killer (NK) cells, and myeloid-derived suppressor cells (MDSCs). The TME also includes surrounding blood vessels, proteins of the extracellular matrix (ECM), and a number of signaling molecules including cytokines and chemokines. Cancer cells along with T regulatory cells (Tregs), MDSCs, adipocytes, and tumor-associated macrophages (TAM) can hinder immune control of tumors by producing and releasing cytokines, such as interleukin-10 (IL-10), chemokines, such as chemokine C-X-C motif ligand 12 (CXCL12), growth factors, such as transforming growth factor beta (TGF-β), matrix remodeling factors, such as collagen, fibronectin, and fibrin, and other soluble factors, such as adenosine, into the TME [26,27]. The final effect consists in a strong immunosuppressive identity in the last phase (elimination) of the cancer immunoediting process of the tumor niche. In this environment, the immune system fails to recognize TAAs and tumor-associated neoantigens because tumor cells have devised ways to escape immune surveillance. In particular, TGF- β and IL-10 mediate an anti-inflammatory response by dampening the activity of tumor suppressor cells, such as cytotoxic T lymphocytes (CTLs) and NK cells, and enhancing the activity of tumor-promoting cells such as Tregs and tumor-associated neutrophils (TANs) [28,29].

As previously mentioned, the main advantage of using oncolytic viruses (OVs) is their ability to modulate the TME rendering it less immunosuppressive [30]. OVs can preferentially infect and kill cancer cells as result of the inhibition of the dysfunctional Type I IFNs signaling [31]; however, their main ability consists in inducing a response from the immune system impaired by the hostile and highly immunosuppressive environment of the tumor milieu. In fact, after a successful tumor infection, an inflammatory reaction is triggered because OVs are able to induce a particular form of apoptosis better known as immunogenic cell death (ICD). During this process, the OV-mediated cancer cell lysis releases TAAs into the microenvironment allowing the immune system to recognize them and to generate a response, breaking down the immuno-editing process. Specifically, TAAs recruit and activate DCs with consequent stimulation of specific lymphocytes, evoking an effective anti-tumor response. Then, the ICD is not sterile, but it triggers the endoplasmic reticulum with the consequent release of some dangerous metabolites called damage-associated molecular patterns (DAMPs) such as calreticulin, ATP, and HMGB1 [32]. Furthermore, ICD mediated by OVs is associated with the release of pathogen-associated molecular patterns (PAMPs) that bind pattern recognition receptors (PRRs) on innate immune cells and function as danger and eat-me signals. The recognition of these key metabolites by the APCs in the tumor microenvironment contributes to trigger an immune response. Therefore, virus-mediated ICD leads to an inflammatory response and a localized cytokine production followed by infiltration of innate immune cells and CTLs that help to shape the TME in a less immunosuppressive manner [33]. Despite the multipower of OVs, all that glitters is not gold because the antitumor- immunity generated by OVs is hampered by the classical anti-viral response from normal cells. The activation of the immune system destroys infected cancer cells but also clears the OV, reducing the therapeutic efficacy [32]. This immunological system has to be manipulated in order to balance the anti-viral response with the anti-tumoral response. This can be obtained by designing OVs that can replicate and spread within tumors quickly to induce maximal anti-tumor effect before clearance [34] or by increasing the recruitment of immune cells, which will kill the infected cells (i.e., tumor cells), potentiating the direct lysis of neoplastic cells by viral infection itself. The latter can be improved by arming viruses with immunostimulatory cytokines, chemokines, or immune-activating ligands and bispecific T-cell engager (BiTE) molecules in order to catch more immune components to the tumor site. In the next sections, we discuss the progress made in arming oncolytic adenoviruses and the successful combinations with other immunotherapy solutions.

### 3.1. Armed Oncolytic Adenoviruses with Immunostimulatory Cytokines and Chemokines

The current scientific trend is to attempt an increase of the antitumoral immune response taking advantages of different agents that can counteract cancer immune escape. Although OVs are able to induce anti-cancer immunity by multiple mechanisms, as described in the previous section, recent updates of clinical trials involving OVs confirm their modest activity as a monotherapy. This can be explained by the inability to optimally infect cancer cells due to (i) neutralizing antibodies, (ii) other antiviral clearance mechanisms, (iii) physical barriers that prevents OVs to reach their entry receptors, or due to viral intrinsic factors such as (iv) engineered cancer-selectivity or transgene expression that can reduce viral fitness and (v) expression of potent transgene(s) that may result in a significant immune response with premature clearance of the OV [35]. In this section, we review the current design strategies to harness the potential of oncolytic adenoviruses for cancer immunotherapy. To effectively trigger the immune response necessary for the removal of tumor cells, it is necessary to not only trigger an immune response but also to recruit immune cells. With this aim, many OVs have been modified to express immunostimulatory transgenes, such as interleukins.

Using immunostimulatory cytokines has become an increasingly promising approach in cancer immunotherapy because they indirectly activate tumor-specific T-lymphocytes capable of rejecting tumor cells from patients with a low tumor burden or because they protect patients from a recurrence of the disease. One of the most promising cytokines for arming oncolytic viruses is granulocyte macrophage colony stimulating factor (GM-CSF) [33,36]. Its pro-inflammatory activity is primarily due its role as a growth and differentiation factor of myeloid lineage cells and the granulocyte and macrophage populations in particular [37]. The ability of GM-CSF to enhance antitumor immunity via a T-cell-mediated mechanism has been potentiated by its local expression by OV, improving DC migration and maturation and eventually improving priming of the T-cell response [38]. Various Ads have been successfully armed with GM-CSF, such as ONCOS-102, currently in a phase I trial in combination with pembrolizumab (NCT03003676). Interesting data in support of the ongoing clinical study mentioned were given by L. Kuryk et al., who observed a synergistic anti-tumor effect in the humanized mice treated with the combination of ONCOS102 and pembrolizumab, as demonstrated by reduced tumor volumes [39].

Originally characterized as a potent inducer of natural killer [40] cell cytotoxic activity, interleukin 12 (IL-12) has been used for arming OVs. IL-12 is now recognized as a key regulator of cell-mediated immune response and a bridge between innate and adaptive immunity [41]. Because of its role as major orchestrator of Th1-type immune response against cancer [40], IL-12 is an attractive protein candidate for cancer therapies [42]. Studies conducted in a rat model of thyroid cancer showed that delivery of IL-12 gene with adenovirus (AdIL-12) was efficacious to elicit systemic anti-tumor immunity, unlike treatment with AdGM-CSF with cells expressing IL-12 or GM–CSF, which elicited only local effects. Chemokines constitute the largest family of cytokines, with approximately 50 endogenous chemokine ligands in humans and mice [43]. These small secreted proteins mediate immune cell trafficking and lymphoid tissue development. Different immune cell subsets migrate into the tumor microenvironment via interaction between chemokines and chemokine receptors, and these populations regulate the tumor immune response in a spatiotemporal manner, thus affecting disease progression and therapeutic results [44]. Different chemokines, such as CCL5 and CCL19, have been expressed in various types of virus; in particular, CCL20 and CCL21 have shown to enhance anti-tumor effects when used to arm Onc.Ads [45,46]. The generation of effective anti-tumor immune responses is a complex process dependent upon the coordinated interaction of various subsets of effector cells. As such, CCL21 and IL-21 are potent activators of the immune system when used together for tumor therapy. Multigene-armed oncolytic adenoviruses are capable of efficiently generating a productive antitumor immune response. Li et al. armed an oncolytic adenovirus with the chemokine (C-C motif) ligand 21 (CCL21) and with Interleukin 21 (IL-21) that was able to induce oncolytic effects and a tumor-specific cytotoxic T-lymphocytes (CTLs) response in vitro [45]. A similar strategy that combined CCL20 and CD40L was adopted resulting in an enhanced growth suppression of TERT-positive tumor cells [46].

### 3.2. Arming OVs with Immune-Activating Ligands and Bispecific T-Cell Engager (BiTE) Molecules

OVs have been shown to exert beneficial immunologic responses, including induction of anti-tumor T-cells and modulation of the tumor microenvironment from Th2 to Th1, which has been suggested to contribute to breakage of tolerance in tumors [47,48,49]. Nevertheless, oncolysis per se is usually not enough for immunologic eradication of advanced tumors and its action could be increased by arming the virus with immune stimulatory molecules. One of the most investigated immune-activating ligands is CD40L because it constitutes an interesting target in cancer immunotherapy because of its ability to stimulate Th1 immunity via maturation of dendritic cells and to drive M2 to M1 macrophage differentiation [50]. CD40 is a member of the tumor-necrosis factor (TNF) receptor family and is expressed on APCs, such as DCs and myeloid cells [51]. APCs greatly increase their antigen-presentation and costimulatory capacity and allow for efficient CD8^+^ CTL priming by signaling through CD40 [52]. In addition, CD40L is expressed on activated CD4^+^ T-cells, B-cells, and NK-cells as well as memory CD8^+^ T-cells [51]. Many OVs and viral vectors armed with CD40L have been tested in clinical [53,54,55,56] and preclinical [50,57,58] settings and have been shown to exert multiple antitumoral activities including tumor growth control, cancer cell apoptosis, induction of T-cell responses, increase in T-effector/T-reg cell ratios, and upregulation of Th1 cytokines. For instance, Pesonen et al. treated nine patients with refractory solid tumors using an OV armed with CD40L (CGTG-401) intratumorally, reporting that 83% of patients showed some disease control and experienced some grade 1 to 2 adverse events. However, induction of a tumor-specific T-cell response was observed in the majority of patients [53]. Furthermore, NG-350A, an Onc.Ad expressing a full-length agonist anti-CD40 antibody at the site of virus replication, is under investigation in a phase I clinical trial (NCT03852511).

The tumor necrosis factor receptor superfamily, member 4 (TNFRSF4), also known as CD134 and OX40 receptor, is another member of the TNFR-superfamily of receptors that have gained interest as therapeutic target molecules for cancer immunotherapy. OX40 is not constitutively expressed on resting naïve T-cells and plays a key role in the survival and homeostasis of effector and memory T-cells, and it regulates the differentiation and function of Foxp3+ Tregs [59]. H. Jiang et al. have recently showed that OX40L-armed Onc.Ad (Delta-24-RGDOX) has a stronger anticancer efficacy compared to its predecessor Delta-24-RGD, triggering a greater tumor-specific lymphocyte activation and a proliferation of TAAs-specific CD8^+^ T-cells in two mouse glioma models [60]. In the same model, a synergistic therapeutic effect was observed by the intra-tumoral injection of Delta-24-RGDOX and an anti-PD-L1 antibody [61]. Therapeutic efficacy of Delta-24-RGDOX has been subsequently evaluated in subcutaneous and intracranial melanomas. Localized treatment of the subcutaneous melanoma inhibited growth of the intracranial ones, suggesting a strong systemic immunity in syngeneic glioma mouse models. Currently, a phase I trial is going on to evaluate the effects of Delta-24-RGDOX treatment in patients with recurrent glioblastoma (NCT03714334) and a phase II trial is evaluating the effects of pembrolizumab together with Ad5-DNX-2401 or Delta-24-RGD (NCT02798406) as reported in Table 1. Oncolytic virotherapy is being evaluated as a therapeutic approach in models for aggressive pediatric brain tumors, such as pediatric high-grade glioma (pHGG) and diffuse intrinsic pontine gliomas (DIPGs), with encouraging results in mouse models [62]. These data led to a phase I/II clinical trial for newly diagnosed diffuse intrinsic pontine gliomas (DIPG) (NCT03178032).

CD40L has also been evaluated in combination with an additional co-stimulatory molecule, named 4-1BBL, to arm OVs [61]. 4-1BBL belongs to the TNFR family and is expressed on activated T-cells. Signaling through 4-1BB/4-1BBL stimulates T-cell expansion, acquisition of effector function, and survival [12]. The virus LOAd703, armed with CD40L and 4-1BBL, was shown to act as a potent immune activator in in vivo xenograft models of human pancreatic cancer. Such a double-armed virus efficiently reduced established tumors and could be combined with gemcitabine for additional effect. Currently, LOAd703 is undergoing two phase I/II clinical trials in patients with pancreatic cancer (NCT02705196) and in patients with pancreatic adenocarcinoma, ovarian cancer, biliary carcinoma, or colorectal cancer (NCT03225989). Finally, another costimulatory molecule that has successfully been used to arm OVs is glucocorticoid-induced tumor necrosis family receptor family-related gene (GITR) [63]. GITR is a modulator of immune response and inflammation; functional testing with specific antibodies showed that only an anti-GITR antibody could inhibit immune suppressive activity of an immunosuppressive T-cell population, T-regulatory cells (T-reg). This population, which expresses both CD4 and CD25, has been implicated in protecting tumors from immune attack and in supporting their growth in mouse models.

BiTE molecules are a novel class of immunotherapeutic agents that can activate T-cells independently of MHC expression to lyse target cells. One arm of the BiTE molecule binds CD3-epsilon on the T-cell receptor, whereas the other arm can bind a defined target antigen. Binding of both arms to their corresponding target antigens triggers T-cell activation leading to target cell lysis by apoptosis [64]. Recently, Freedman et al. armed Onc.Ads to express a BiTE molecule that binds to the epithelial cell adhesion molecule (EpCAM) overexpressed on target cancer cells (EnAd-SA-EpCAM). Remarkably, EnAd-SAEpCAM could activate endogenous T-cells within the immune-suppressive microenvironment of liquid cancer biopsies (malignant peritoneal and pleural exudates) and exhibited killing of endogenous tumor cells without addition of exogenous T-cells [65]. Another Onc.Ad that incorporates BiTE molecules is NG-641, which expresses a fibroblast activation protein (FAP)-targeting bispecific T-cell activator (FAP-TAc) antibody together with an immune enhancer module (CXCL9/CXCL10/IFNα) [65]. NG-641 is able to eradicate tumor-associated stromal fibroblasts in order to reduce tumor growth and stimulate anti-cancer immune responses, even in tumors poorly infiltrated by immune cells. Therefore, FAP-TAc allows the activation of T-cells and decreases the tumor-associated fibroblasts in tumor stroma. In addition, in order to enhance the potential for activity in tumors poorly infiltrated by immune cells, NG-641 was designed to additionally encode the immune enhancer molecules IFNα and CXCL9/10. A phase I clinical trial to characterize the safety and tolerability of NG-641 in patients with metastatic or advanced epithelial tumors is ongoing (NCT04053283).

## 4. Oncolytic Adenoviruses and Immunotherapy

The recent successes of anti-tumoral immunotherapy approaches, such as ICIs and chimeric antigen receptor T-cell (CAR-T) therapy, have revolutionized cancer treatment, improving efficacy and extending treatment to a larger proportion of cancer patients [66]. However, due to the high heterogeneity of cancer, poor tumor cell targeting, and the immunosuppressive status of TME, combinatorial agents are required to obtain more effective and consistent therapeutic responses in a wide range of cancers. As has been well demonstrated by different published studies [67,68], OVs, for their inherited cancer-killing abilities, can be used as initial priming agents to overcome TME-associated immunosuppression, generating a milieu conducive to the efficacy of subsequent ICIs immunotherapies in brain and breast cancers. These findings have encouraged many scientists to think that the adjuvant-like properties of OVs, imbedded within the immunological responses driven by their therapeutic administration, could be exploited to enhance the efficacy of cancer immunotherapies. V. Cervera-Carrascon et al. observed outstanding results, reporting complete remission and survival in a syngeneic mouse model of melanoma treated with an Onc.Ad expressing TNFα and IL-2, with a subsequent anti-PD-1 therapy in a prime and boost manner [69]. This successful strategy induced pro-inflammatory danger signals in the tumor microenvironment and led to effective recruitment and stimulation of anti-tumor T-cells, whose exhaustion was prevented by the anti-PD-1 antibody. These results set the stage for clinical evaluation of TNF-alpha and IL-2 expressed oncolytic adenovirus (TILT-123) in melanoma patients treated with the anti-PD-1 antibody [70]. A recent clinical trial is evaluating TILT-123 in melanoma patients receiving adoptive cell therapy with tumor-infiltrating lymphocytes (NCT04217473). Results obtained by Feola et al. demonstrated that OV-based cancer vaccine can significantly improve the response rate to ICIs antibodies in the context of immunogenic and non-immunogenic tumors [4]. They observed that anti-PD-L1 therapy in combination with PeptiCRAd [71] significantly reduced the melanoma growth of and increased the response rate to ICIs in a mouse model of melanoma. The combined approach resulted in increased non-exhausted antigen-specific T-cells within the tumor in comparison to anti-PD-L1 monotherapy. Belcaid Z. et al. previously showed that Delta24-RGD affects local innate immune cells in glioblastoma by inducing phenotypic skewing of pro-tumor M2-like macrophages toward an anti-tumor M1-like phenotype, therewith inducing a pro-inflammatory environment. They also observed that a low-dose Delta24-RGD therapy effectively sensitizes murine gliomas models to sequential anti-PD-1 therapy. This combined solution synergizes to overcome adaptive immune resistance induced by intra-tumoral PD-1 expressing CD8^+^ T cells, leading to an effective IFNγ-mediated antitumor immune response and a long-term cure of glioma-bearing mice [72]. The virus LOAd703 armed with TMZ-CD40L and 4-1BBL was shown to act as a potent immune activator in in vivo xenograft models of human pancreatic cancer. Such a double-armed virus efficiently reduced established tumors and could be combined with gemcitabine for additional effect.

In the process of writing this review, we searched for OVs combined with ICIs clinical trials on the clinicaltrials.gov website; as shown in Table 1, of the work published by Tao Shi [66], numerous combinations of different types of OVs with ICIs have been investigated in various clinical trials, among which the anti-PD-1/PD-L1 and anti-CTLA-4 combination therapies had promising results in the objective response rate and overall survival. A single trial investigated a combination of Ad-p53 with oral pembrolizumab in patients with unresectable, refractory liver metastases of colorectal carcinoma (CRC) and other solid tumors, including primary hepatocellular carcinoma (HCC), and an additional solution of Ad-p53 combined with nivolumab in recurrent head and neck squamous cell cancer (HNSCC) was terminated. In addition, on clinicaltrials.gov, two additional phase I/II clinical trials with Onc.Ads were active and not yet recruiting, respectively, (as indicated in Table 1), suggesting growing interest and encouraging patient outcomes. No active phase III clinical trials were found.

## 5. Limitations of Onc.Ads

To date, immunotherapy confirms its powerful efficacy against cancer because it can be long-lasting because of the generation and maintenance of tumor-specific memory T-cells [73]. Among the several immunotherapeutic solutions, OVs remain an appealing and pluripotent tool. First, they offer the chance of transgene delivery amplifying the local transgene expression at the tumor site, therefore reducing systemic adverse effects of the transgene, relevant in case of cytokines. With an OV approach, the virus-induced expression of a cytokine triggers immunological reactions, in addition to the ones the virus itself induces. Using Onc.Ads to express immune-modulatory therapeutics has several advantages over the combination of Onc.Ads with exogenous immune-therapeutics. Cytokines usually have short half-lives and act over short distances, which is why repeated injections of high doses are required to achieve meaningful anti-tumor effects. In addition, Onc.Ads, armed with immunostimulatory cytokines, at least in theory, will ensure an immune response at least as long as the virus persists in the tumors. Hence, cytokines or other immune-modulatory therapeutics encoded by Onc.Ads within the tumor milieu will be more effective, less toxic, and cost-effective [1]. Second, the ICD triggered by Onc.Ads recruits immune cells to the tumor and releases TAAs and immunological danger signals contributing to modulate the TME towards a less immunosuppressive status. The change from “cold” into “hot” tumor increases the opportunities to develop tumor-specific responses and epitopes spreading [74]. Although Onc.Ads provide a versatile and advantageous platform for the expression of immunotherapeutics, they meet a critical barrier that limits their antitumor activity. The immune system is one of the classically suggested limiting factors [75]. Antibody-mediated neutralization of non-enveloped viruses and complement activity for enveloped ones can reduce the efficacy of systemic administration, but even this assumption is starting to be examined [1,76]. Based on clinical results, there is no correlation between the anti-virus neutralizing antibody titers and antitumor effects [77,78]. Interestingly, a novel strategy has been suggested to convert an unfavorable immune response into an anticancer immunotherapy by tumor retargeting of antiviral antibodies [79]. Besides the antiviral immunity barrier, another crucial limitation for OVs is represented by the dense extracellular matrix, stromal barriers, or hypoxic conditions, which can limit their replication and spreading [80,81,82]. Several studies have tried to overcome this limitation by arming OVs with stromal-degrading enzymes including collagenase, hyaluronidase, and decorin [83,84,85].

Considering the Onc.Ads in the larger set of Ad vectors, we can surprisingly appreciate their utility during the COVID-19 pandemic. A recent work has been published about an Ad5-vectored COVID-19 vaccine that is tolerable and immunogenic at 28 days post-vaccination, even if these findings need further investigation [86].

## 6. Future Outlook: A Challenge for Oncolytic Viro-Immunotherapy

Cancer cell metabolism is strictly regulated by the TME and cells tend to use aerobic glycolysis to support biosynthetic pathways even in the presence of oxygen [87]. Like tumor cells, activated T-cells depend on glycolysis to support cellular proliferation and effector functions in contrast to naïve or memory T-cells, which mainly engage mitochondrial respiration to perform their biological functions [88]. Tumor cells, TILs, and other immune or stromal cells within the tumor milieu share similar biosynthetic pathways and compete for limited nutrients; in addition, certain metabolites produced within the TME may dampen antitumor immunity [89,90]. Human cancers are able to subvert this metabolic stress condition of the TME, escaping immunosurveillance from the host immune system. The deprivation of nutrients and the exposure to oxygen cause a downregulation of class I MHC, thereby escaping recognition and rejection by anti-tumor T-cells [91].

A novel component in the TME that alters cancer cell metabolism is the microbiome [92]. Evidence has shown that perturbations in the microbiome composition have intricate connections with neoplastic disease [93]. An altered microbiome composition can promote or inhibit tumorigenesis through the modification of the immune response and microbiome-derived metabolites, such as estrogen [94], secondary bile acids, genotoxin, and short-chain fatty acids [95]. Surprisingly, the microbiome is able to affect tumor cells’ metabolism by maintaining a healthy barrier, inducing inflammation, and producing genotoxins and bacterial metabolites with different features. Investigating the bacterial communities and metabolite-related bacteria that positively influence the metabolically-stressed TME could be a promising research route.

Taken together, we think that one of the next goals of viro-immunotherapy could be to develop new strategies useful for a metabolic reprogramming, in order to shape the TME for an effective antitumor T-cell immunity. Understanding metabolic communication between tumor cells and other cellular components (e.g., white blood cells) within the TME could have great therapeutic value, and targeting the metabolic crosstalk may directly affect the biological activity of tumor cells and immune cells. In addition, considering the microbiome effects on tumor cell metabolism, we believe that identifying the bacteria or bacterial-derived metabolites connected with a metabolic stress condition within the TME could contribute to the design of novel OVs.

## Figures and Tables

**Table 1:** Current clinical trials including oncolytic viruses (OVs) combined with immune checkpoint inhibitors. Clinical trials reported as completed are not listed.

OV Type	Genetic Modification	Checkpoint Inhibitor	Indication	Clinical Phase	NCT Number
Herpes simplex virus 1	Deletions in ICP34.5 and ICP47 and transgenic expression of GM-CSF	Pembrolizumab (anti-PD1)	Unresectable Stage IIIB–IV melanoma	III	NCT02263508
T-VEC		Nivolumab (anti-PD1)	Lymphomas and some rare cutaneous tumors	II	NCT02978625
		Pembrolizumab	Advanced melanoma progressed on anti-PD1/L1 based therapy	II	NCT02965716
		Pembrolizumab	Metastatic squamous cell carcinoma of the head and neck	I	NCT02626000
		Ipilimumab (anti-CTLA4)	Melanoma	I/II	NCT01740297
		Atezolizumab (anti-PDL1)	Breast cancer	I	NCT03802604
		Ipilimumab and nivolumab	Before surgery of localized breast cancer	I	NCT04185311
Vaccinia virus Pexa-Vec	TK deletion and expression of GM-CSF and β-galactosidase	Ipilimumab	Metastatic solid tumors	I	NCT02977156
		Durvalumab (anti-PD1)-Tremelimumab	CRC	I/II	NCT03206073
		(Anti-CTLA4) nivolumab	HCC	I/II	NCT03071094
		Cemiplimab (anti-PD1)	RCC	I	NCT03294083
Vesicular stomatitis virus (VSV)	Engineered to express Na^+^/I^−^ symporter (NIS) and human	Avelumab	Refractory solid tumors	I	NCT02923466
	Interferon Beta (VSV-IFNβ-NIS)	Pembrolizumab	Refractory NSCLC and HCC	I	NCT03647163
Reovirus reolysin	None	Nivolumab	Relapsed/refractory multiple myeloma	I	NCT03605719
Adenovirus (Ad) ONCOS-102	Onc.Ad expressing GM-CSF	Pembrolizumab	Advanced or unresectable melanoma	I	NCT03003676
CG0070	Onc.Ad with a tumor specific promoter expressing GM-CSF	Pembrolizumab	NMIBC	II	NCT04387461
Ad-p53	Ad. expressing p53	Pembrolizumab	HNSCC	I/II	NCT02842125
		PD-1/PD-L1 Inhibitors	Lymphoma	II	NCT03544723
Ad-MAGEA3	Ad. expressing MAGE-A3 with MG1-MAGEA3	Pembrolizumab	NSCLC	I/II	NCT02879760
		Pembrolizumab	Metastatic melanoma squamous cell skin carcinoma	I	NCT03773744
Ad5-DNX-2401 or Delta-24-RGD	Ad. expressing an Integrin-binding RGD-4C motif	Pembrolizumab	GBM and GS	II	NCT02798406

CRC: colon rectal cancer; HCC: hepatocellular carcinoma; RCC: renal cell carcinoma; NSCLC: non-small cell lung cancer; NMIBC: non-muscle invasive bladder cancer; HNSCC: head and neck squamous cell carcinoma; MG1-MAGEA3: MG1 maraba oncolytic virus expressing melanoma-associated antigen 3 (MAGEA3); GBM: glioblastoma; GS: gliosarcoma.
